# Cytokinin as a positional cue regulating lateral root spacing in *Arabidopsis*

**DOI:** 10.1093/jxb/erv252

**Published:** 2015-05-27

**Authors:** Ling Chang, Eswarayya Ramireddy, Thomas Schmülling

**Affiliations:** ^1^Institute of Biology/Applied Genetics, Dahlem Centre of Plant Sciences (DCPS), Freie Universität Berlin, Albrecht-Thaer-Weg 6, D- 14195 Berlin, Germany; ^2^ Present address: Hubei Collaborative Innovation Center for Green Transformation of Bio-Resources, College of Life Science, Hubei University, Wuhan 430062, China

**Keywords:** *Arabidopsis thaliana*, cytokinin, lateral root, lateral root spacing, root branching, root system architecture.

## Abstract

The root systems of plants have developed adaptive architectures to exploit soil resources. The formation of lateral roots (LRs) contributes to root system architecture. Roots of plants with a lower cytokinin status form LR primordia (LRP) in unusually close proximity, indicating a role for the hormone in regulating the positioning of LRs along the main root axis. Data obtained from cytokinin-synthesis mutants of *Arabidopsis thaliana* combined with gene expression analysis indicate that cytokinin synthesis by IPT5 and LOG4 occurring early during LRP initiation generates a local cytokinin signal abbreviating LRP formation in neighbouring pericycle cells. In addition, IPT3, IPT5, and IPT7 contribute to cytokinin synthesis in the vicinity of existing LRP, thus suppressing initiation of new LRs. Interestingly, mutation of *CYP735A* genes required for *trans*-zeatin biosynthesis caused strong defects in LR positioning, indicating an important role for this cytokinin metabolite in regulating LR spacing. Further it is shown that cytokinin and a known regulator of LR spacing, the receptor-like kinase ARABIDOPSIS CRINKLY4 (ACR4), operate in a non-hierarchical manner but might exert reciprocal control at the transcript level. Taken together, the results suggest that cytokinin acts as a paracrine hormonal signal in regulating root system architecture.

## Highlight

Cytokinin synthesis gene expression patterns and mutant phenotypes show the relevance of cytokinins in generating a paracrine signal to regulate lateral root spacing, which is important in shaping root system architecture.

## Introduction

Lateral roots (LRs) form the main part of plant root systems and are important to optimize the ability of a root system to acquire soil nutrients and water. In *Arabidopsis*, LRs are formed along the main root axis from xylem pole pericycle cells (PCs) according to certain regularities, such as that lateral root primordia (LRP) usually do not form adjacent or opposite to each other (De Smet *et al.*, 2006; Dubrovsky *et al.*, 2006; reviewed by Dastidar *et al.*, 2012). Xylem pole PCs are developmentally primed to form competent sites for LR formation along the longitudinal axis. This pre-pattern is established by an endogenous clock-like mechanism, termed the LR clock (Moreno-Risueno *et al.*, 2010). However, not all competent cells develop into LRP or LRs and the mechanisms patterning the local spacing of LRP remain largely uncharacterized. It appears that auxin has a role in regulating the distance between two successive LRP through the BDL/IAA12-MP⁄ARF5 signalling module (De Smet *et al.*, 2010) and transcriptional regulators named PLETHORA3 (PLT3), PLT5, and PLT7 (Hofhuis *et al.*, 2013). Furthermore, the receptor-like kinase *Arabidopsis* CRINKLY4 (ACR4) has been reported to act non-cell autonomously to prevent neighbouring PCs from lateral root initiation (LRI) (De Smet *et al.*, 2008). It has been hypothesized that members of the signalling peptide family GOLVEN (GLV)/ROOT GROWTH FACTORS/CLE-LIKE interact with ACR4 to initiate a signalling cascade (Meng *et al.*, 2012; Fernandez *et al.*, 2013).

Cytokinins are another class of plant hormones playing a crucial role in regulating LR formation and growth, acting as a negative regulator of early processes by preventing the establishment of an auxin gradient required for LR formation (Laplaze *et al.*, 2007; Marhavý *et al.*, 2011). The cytokinin metabolism and signalling pathways of *A. thaliana* have been elucidated and comprise ~60 genes (reviewed by Sakakibara, 2006; Werner and Schmülling, 2009; Kieber and Schaller, 2014). In brief, the first and rate-limiting step in cytokinin biosynthesis is catalysed by isopentenyl transferases (IPT), and the subsequent formation of *trans*-zeatin (*t*Z) metabolites from isopentenyl adenine (iP) metabolites is catalysed by hydroxylase enzymes (cytochrome P450 CYP735A), encoded by two predominantly root-expressed genes (Takei *et al.*, 2004*a*; Kiba *et al.*, 2013). The final step in the synthesis of iP- and *t*Z-type cytokinins is the release of active cytokinins from their nucleotide precursor forms by cytokinin nucleoside 5′-monophosphate phosphoribohydrolases, called LONELY GUY (LOG) (Kurakawa *et al.*, 2007). Cytokinin breakdown is catalysed in a single step by cytokinin oxidase/dehydrogenase (CKX) enzymes (Werner *et al.*, 2006). All of these metabolic enzymes are encoded in *Arabidopsis* by gene families with several members. The cytokinin signal is perceived through three membrane-located histidine kinase receptors (named AHK2, AHK3, and CRE1/AHK4) (Inoue *et al.*, 2001; Yamada *et al.*, 2001) and transmitted by phosphotransmitter proteins (AHPs) (Hutchison *et al.*, 2006) to the nucleus, where type-B response regulators (type-B ARRs) activate downstream genes specifying cytokinin action (Mason *et al.*, 2005). In *Arabidopsis* one member of the AHP family, AHP6, lacks the canonical phospho-accepting His residue and acts as a negative regulator of cytokinin signalling (Mähönen *et al.*, 2006).

First hints that cytokinin could provide positional information for LRs came from an analysis of cytokinin-deficient *CKX* transgenic plants that showed defects in LR spacing (Werner *et al.*, 2003). In addition, a higher LR density has been noted repeatedly on plants with a lower cytokinin content or signalling (Werner *et al.*, 2003, 2010; Mason *et al.*, 2005; Miyawaki *et al.*, 2006; Riefler *et al.*, 2006; Laplaze *et al.*, 2007; Heyl *et al.*, 2008; Bielach *et al.*, 2012; Chang *et al.*, 2013). More specifically, local ectopic expression of a *CKX* gene in lateral root founder cells (LRFCs) caused a higher density of LRs. This indicated that LRFCs could be the source of a cytokinin signal (Laplaze *et al.*, 2007) that might be perceived in the zones of high cytokinin sensitivity in the vicinity of LRP (Bielach *et al.*, 2012). Here, the role of cytokinin in controlling LR positioning was analysed in more detail, and relevant cytokinin metabolism and signalling genes identified. It is proposed that inhibitory concentrations of cytokinin originate in and around existing LRP, and their establishment requires *IPT* and *LOG4* genes. The relationship between this pathway and the ACR4 pathway in controlling rhizotaxis is also discussed.

## Materials and methods

### Plant material and growth conditions

All *A. thaliana* plants used in this study were of the Columbia (Col-0) ecotype. Seeds of *acr4* (*acr4-2*, *acr4-2 crr2-2 acr4-2 crr4*; De Smet *et al.*, 2008), *ipt* (*ipt3-2*, *ipt5-2*, *ipt7-1*, *ipt3-2 ipt5-3*, *ipt3-2 ipt7-1, ipt3-2 ipt5-3 ipt7-1*; Miyawaki *et al.*, 2006), *log4-3* (Kuroha *et al.*, 2009), and *cyp735a1-1, cyp735a2-2, cyp735a1-1 cyp735a2-2* (Kiba *et al.*, 2013) mutants were kindly provided by Ive De Smet, Tatsuo Kakimoto, and Hitoshi Sakakibara, respectively. The marker gene lines *pCKX:GUS* (Werner *et al.*, 2003), *pIPT:GFP* (Takei *et al.*, 2004*b*), *pLOG:GUS* (Kuroha *et al.*, 2009), *pCYP735A2:GUS* (Kiba *et al.*, 2013), *ACR4::H2B:YFP* (Gifford *et al.*, 2003), *CycB1;1:GUS* (Beeckman *et al.*, 2001), *TCSn:GFP* (Zürcher *et al.*, 2013), and *DR5:GUS* (Ulmasov *et al.*, 1997) were obtained from Hitoshi Sakakibara, Gwyneth C. Ingram, Laurent Laplaze, Bruno Müller, and the Nottingham Arabidopsis Stock Centre, respectively. *35S:CKX1*, the type-B *ARR* gene mutant lines (*arr1-3 arr10-5*, *arr1-3 arr11-2*, *arr1-3 arr12-1*, *arr10-5 arr12-1*, *arr11-2 arr12-1*), and the cytokinin receptor double mutants *ahk2-5 ahk3-7*, *ahk2-5 cre1-2*, and *ahk3-7 cre1-2* were described previously (Werner *et al.*, 2003; Mason *et al.*, 2005; Riefler *et al.*, 2006).

Seeds were surface sterilized and plated on solid medium (half-strength Murashige and Skoog [MS] medium, 1% sucrose, and 0.9% agar, pH 5.7) and stratified at 4°C for 3 d in the dark before germination. After stratification, all plates were placed vertically under white light (~100 µmol m^−2^ s^−1^) in long-day conditions (16h light/8h darkness) at 22°C. For cytokinin treatment, seedlings were germinated and grown on half-strength MS medium with or without 6-benzylaminopurine (BA) for 11 d.

### Microscopic analyses

Histochemical staining for β-glucuronidase (GUS) activity was performed essentially according to Jefferson *et al.* (1987) as modified by Hemerly *et al.* (1993). Primary root length was measured on digital images of the plates using ImageJ 1.40 software (http://rsb.info.nih.gov/ij/). For recording the GUS staining pattern and LRP organization, samples were cleared and mounted according to Malamy and Benfey (1997). All samples were analysed by differential interference contrast microscopy (Zeiss Axioskop). The number of PCs between neighbouring LRP was counted in a shootward direction beginning with the LRP closest to the primary root tip.

For fluorescence microscopy, seedlings were mounted in water under glass coverslips for yellow fluorescent protein (YFP) or green fluorescent protein (GFP) signal analysis using a Leica TCS SP5 confocal laser scanning microscope. For YFP fusion protein, a wavelength of 517nm and a filter of 526–568nm were used for excitation and signal analysis, respectively. The fluorescence signal of GFP was analysed with a 488nm argon laser in combination with a 500–530nm filter set.

### Quantitative real-time reverse transcription PCR

Total RNA was extracted from roots of 11-d-old *Arabidopsis* plants with the TRIzol method as described by Brenner *et al.* (2005). cDNA synthesis, quantitative PCR (qPCR), and data analysis were performed according to Werner *et al.* (2010). *UBC10* (At5g53300) and *PDF1* (At3g25800) were used as reference genes to normalize expression levels. Primer sequences for all genes analysed are listed in Supplementary Table S1.

## Results

### Positioning of LRP is altered in cytokinin-deficient plants

**Fig. 1.**
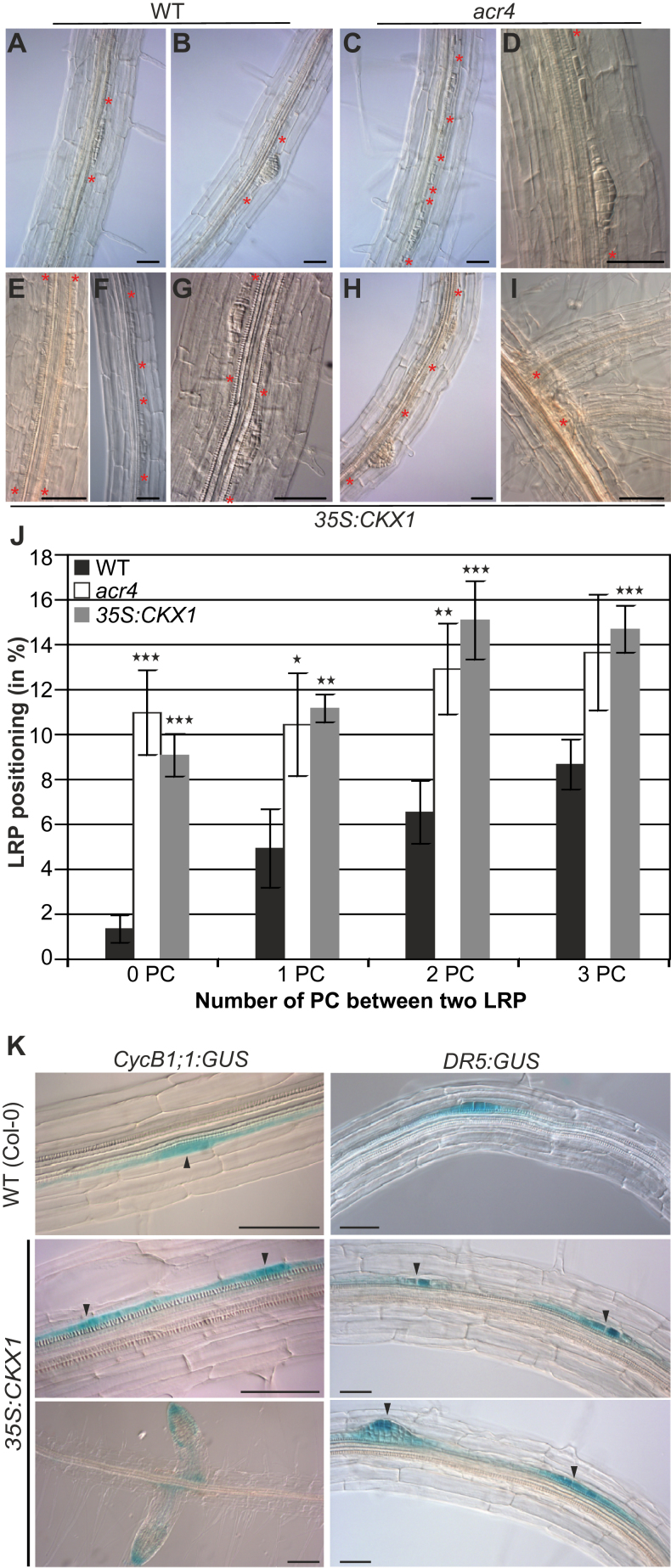
LR spacing is altered in cytokinin-deficient plants. (A-I) No proximal LRP and LRs were observed in wild type (A, B) whereas LRP and LRs in close proximity were observed in *acr4* (C, D) and *35S:CKX1* (E-I). Red asterisks indicate borders of LRP or emerged LRs. (J) Proportion of LRP separated by different PC number in 11-d-old wild-type, *acr4*, and cytokinin-deficient *35S:CKX1* plants. n (number of roots analysed) = 15. (K) Expression of the *CycB1;1:GUS* and *DR5:GUS* marker genes indicates division of neighbouring PCs in *35S:CKX1* plants but not in wild-type plants and a normal auxin pattern in aberrantly positioned LRP and LRs. Black arrowheads indicate LRP. Bar size in (A-I) and (K) is 50 µM. Significance of differences in (J) was analysed by two-tailed Student’s *t*-test. *P < 0.05; **P < 0.01; ***P < 0.001. Error bars indicate SEM (this figure is available in colour at *JXB* online).

First, LR spacing in cytokinin-deficient *35S:CKX1* transgenic *Arabidopsis* seedlings (Werner *et al.*, 2003) was compared with *acr4* mutant seedlings known to have a LR positioning defect (De Smet *et al.*, 2008) and wild-type seedlings under the same growth conditions. In wild-type roots almost no LRP or emerged LRs initiated from immediately adjacent or opposite sites (Fig. 1A,B). Among 373 LRI events in 15 wild-type roots, only five LRP (1.3±0.6%) were located in immediate proximity (Fig. 1J). In contrast, in *acr4* mutant plants LRP initiated from adjacent or opposite sites (Fig. 1C,D), which is consistent with previous results (De Smet *et al.*, 2008). In total, 39 LRP out of 348 LRI events (11.0±1.9%) were formed from these aberrant positions (Fig. 1J). Similarly, 76 LRP out of 811 LRI events (9.1±0.9%) were located immediately adjacent or opposite to each other in *35S:CKX1* plants (Fig. 1J). There was phenotypic variability in the defective spacing pattern. In some instances stretches of several PCs showed cell division activity (Fig. 1E) and closely spaced LRP were formed either on the longitudinal axis (Fig. 1F) or on the opposite side of the axis (Fig. 1G). Closely spaced LRP mostly belonged to the same developmental stage or differed by just one stage (according to Malamy and Benfey, 1997), indicating that they were induced at a similar time (Fig. 1F,G). Albeit less frequent, cytokinin-deficient plants also sometimes formed clusters of closely spaced LRP of different growth stages (Fig. 1H). Rarely, several closely spaced LRP emerged and appeared as clustered LRs (Fig. 1I). However, the proportion of emerged neighbouring LRs was low, suggesting that most of the LRP at aberrant positions arrest during development and do not contribute much to the overall root architecture. Notably, the primary roots of *acr4* mutants were only about 15% shorter than those of wild type (data not shown) and those of *35S:CKX1* transgenic plants were even longer (Werner *et al.*, 2003), indicating that the shorter distance between LRP is not correlated with the length of the primary root.

To study the reach of defective LRP positioning, the percentage of LRP separated by zero, one, or up to three PCs was determined in roots of wild-type, *acr4*, and *35S:CKX1* plants (Fig. 1J). The largest difference between wild type and the mutants was found for a distance of zero PCs, i.e. directly neighbouring cells, and an increased frequency was also observed in the mutants for a LRP distance of up to three PCs (Fig. 1J). In wild type, a total of 21.5% of the LRP had a neighbouring LRP within this distance, while it was 48.0% and 50.0% for *acr4* and *35S:CKX1*, respectively. All other LRPs had a neighbouring LRP at a larger distance. Together, the LR spacing defects in *acr4* mutants and *35S:CKX1* plants occurred at a similar frequency and with a similar spatial distribution.

In order to analyse cell division activity during LRP formation, expression of the *CycB1;1:GUS* reporter marking dividing LRFCs (Casimoro *et al.*, 2001) was compared between wild type and cytokinin-deficient plants. In wild type, GUS staining was observed only in the LRFCs but not in the neighbouring PCs. In contrast, in cytokinin-deficient roots neighbouring PCs could be activated simultaneously to become LRFCs, which is indicated by the cell division activity following their specification (Fig. 1K). To investigate whether closely spaced LRP establish a normal auxin pattern, the auxin reporter *DR5:GUS* was analysed in the *35S:CKX1* background. *DR5:GUS* marked founder cells and subsequent primordium formation in both wild type and neighbouring LRPs in *35S:CKX1* plants and displayed similar expression patterns (Fig. 1K). Apparently closely spaced LRP establish a normal cellular division and auxin pattern and lack growth defects seen in other mutants showing spatially altered LRI (Hofhuis *et al.*, 2013).

### Cytokinin metabolism and signalling genes are involved in the positioning of LRP

**Fig. 2.**
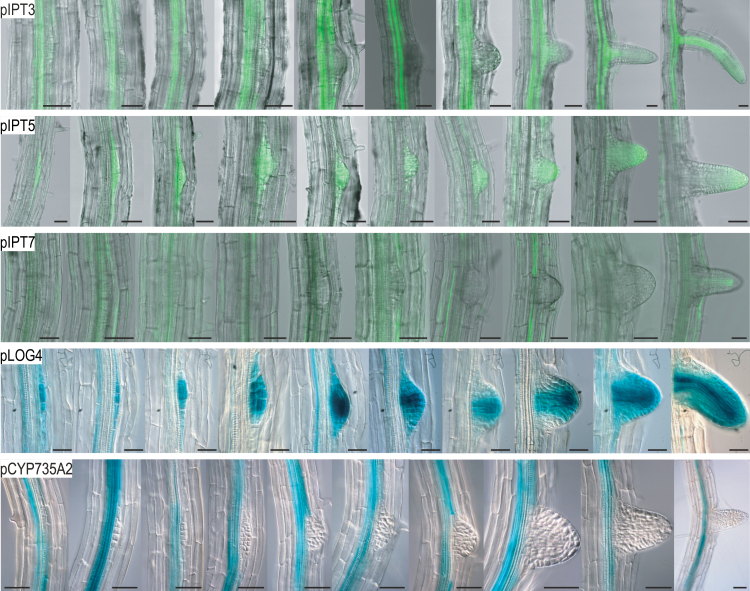
Spatio-temporal expression of selected cytokinin synthesis genes during LR development. A developmental sequence of the expression pattern of promoters of cytokinin metabolism genes is shown from left to right starting with stage I LRP to emerged LR. The respective promoter is indicated in the upper left corner of each picture series. Three-day-old seedlings were stained with GUS reaction buffer for 1h and cleared. GFP expression was analysed in 5-d-old seedlings using a confocal laser scanning microscope. Part of the root staining pattern reporting expression of the cytokinin metabolism genes shown here have been published before (Werner *et al.*, 2003; Takei *et al.*, 2004*b*; Kuroha *et al.*, 2009; Kiba *et al.*, 2013). Scale bars is 50 μM (this figure is available in colour at *JXB* online).

The LR spacing phenotype of cytokinin-deficient plants suggests that the normally occuring inhibition of LRP formation in the vicinity of a LRP that has started to form is abolished. In order to study the spatial organization of the signal generation mediating this inhibition, expression patterns and functions of cytokinin synthesis genes were analysed. The expression of several cytokinin synthesis genes, both *IPT* and *LOG* genes, as well as of cytokinin-degrading *CKX* genes has been reported to occur during LR formation (Werner *et al.*, 2003; Miyawaki *et al.*, 2004; Takei *et al.*, 2004b; Kuroha *et al.*, 2009; Parizot *et al.*, 2010; Kiba *et al.*, 2013). The spatio-temporal expression pattern of these genes during different stages of LR formation was studied in more detail because it has been reported in most cases only for one or two stages. A particularly intriguing expression pattern was shown by the *IPT5*, *LOG4*, and *CKX6* genes, which were switched on very early during formation of LRP in stage I similar to *DR5:GUS* (Figs 1K and 2; Supplementary Fig. S1). During further LRP development *IPT5* and *LOG4* were expressed in most cells until emergence. In the emerged LR the expression of *IPT5* became confined to the root tip and *LOG4* was strongly expressed in provascular tissue and the LR meristem (Fig. 2). In contrast, *CKX6* expression was observed in the vasculature and only in stage I LRP but not in the following stages (Figure S1). The *pIPT3:GFP* and *pIPT7:GFP* reporter did not show activity during early LR development. *IPT3* was expressed in the PCs and the basal stele of young LRs, *IPT7:GFP* expression was confined principally to the vascular stele of the primary root (Fig. 2). Expression of reporter genes for *LOG3*, *CKX1* and *CKX5* became only visible in the vasculature and at the basis of the emerged LR (Supplementary Fig. S1). Also for *pCYP735A2:GUS*, no expression was seen in the primordium itself but there was a variable pattern and strength of expression in PCs and vascular tissue on either side of the existing primordia (Fig. 2). The expression pattern of *pCYP735A1:GUS* could not be analysed because of its low expression level (Takei *et al.*, 2004*a*; Kiba *et al.*, 2013). The gene expression analysis showed a specific pattern of cytokinin synthesis gene expression and pointed to the *IPT5* and *LOG4* genes as being possibly particularly relevant in creating a source of cytokinin in the young primordia. Their expression could establish an inhibitory field preventing LRI in neighbouring cells.

**Fig. 3.**
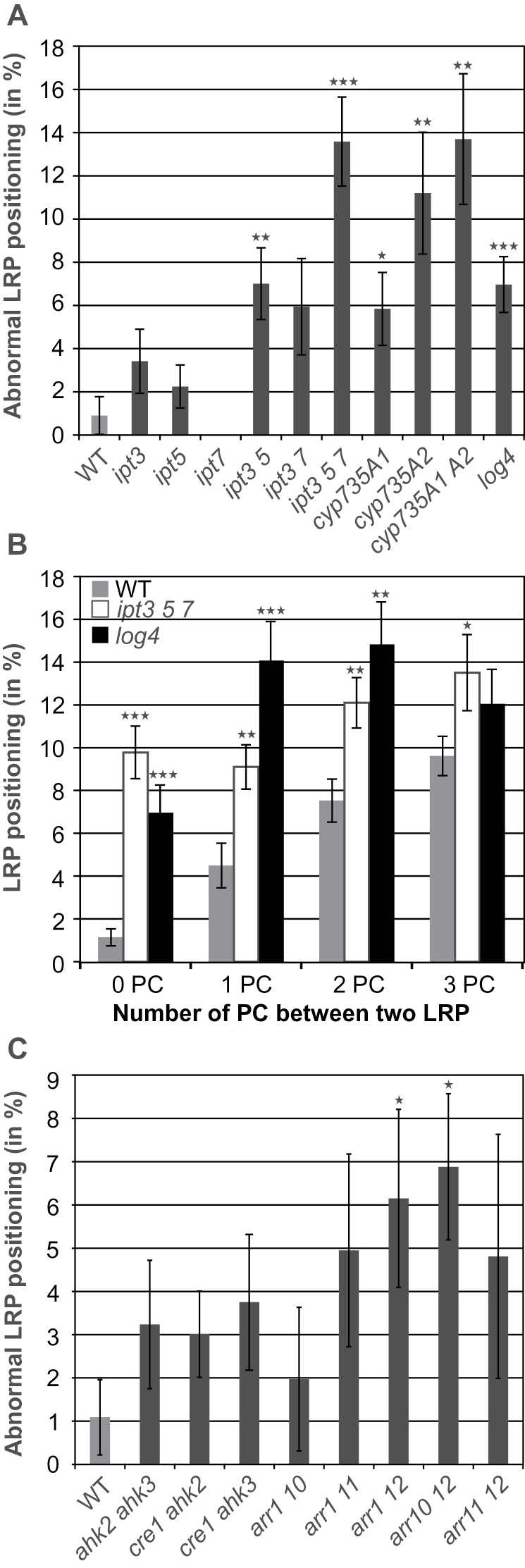
A decrease in cytokinin content or signalling increases the frequency of defects in positioning of LRP. (A) Percentage of LRP positioned immediately adjacent to each other in 11-d-old seedlings of cytokinin-synthesis mutants. n (number of roots analysed) = 12–16. (B) Proportion of LRP separated by zero to three PCs in wild type (WT; n = 30) and the cytokinin-synthesis mutants *ipt3 5 7* and *log4*. n = 15–17. (C) Percentage of LRP positioned immediately adjacent to each other in 11-d-old seedlings of cytokinin-signalling mutants. n = 11–14. Significance of differences in (A-C) was analysed by two-tailed Student’s *t*-test. *P < 0.05; **P < 0.01; ***P < 0.001. Error bars indicate SEM.

To further investigate the role of cytokinin synthesis genes in LR positioning, a detailed analysis was performed using single and higher order mutants. Single *ipt* mutants showed only weak (*ipt3*, *ipt5*) or no (*ipt7*) LRP positioning defects (Fig. 3A). The double mutants *ipt3 5* and *ipt3 7* showed an increased proportion of abnormally positioned LRP compared to wild type but only the difference seen for *ipt3 5* was statistically significant. The proportion of misplaced LRP increased further to 13.6±2.0% (57 out of 416 LRP in 12 roots) in *ipt3 5 7* roots (Fig. 3A). The misplaced LR in *ipt* mutants displayed a similar pattern to that shown in *35S:CKX1* plants and included different developmental stages ranging from LRP to emerging LR (Supplementary Fig. S2). The *log4* mutant also exhibited LR spacing defects (7.0±1.4%) (Fig. 3A).

Single and double mutants of *CYP735A* genes also showed an abnormal LRP positioning phenotype. The *cyp735a1* and *cyp735a2* single mutant phenotypes were enhanced in the *cyp735a1 a2* double mutant, with 13.9±3.0% of the LRI events located in the immediate vicinity (Fig. 3A). This double mutant contained less than 5% of wild-type levels of *t*Z-type cytokinins but a 2-fold higher level of iP-type cytokinins (Kiba *et al.*, 2013). Thus the mutant phenotype indicates a particular functional relevance of *t*Z in regulating LRP spacing.

**Fig. 4.**
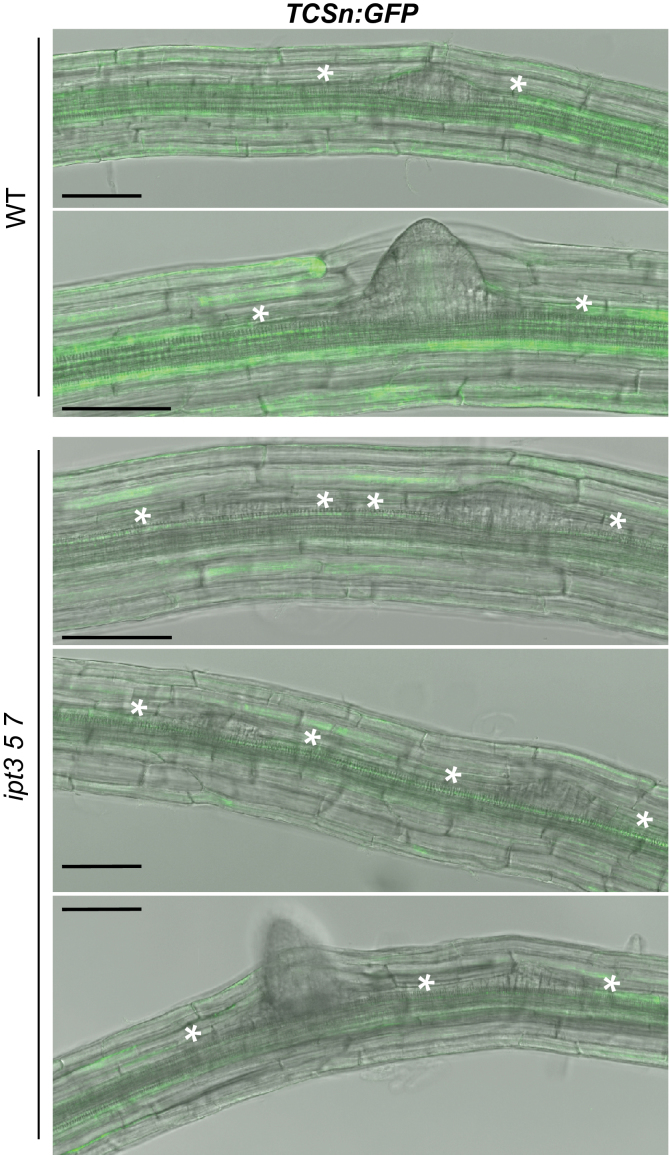
Expression of *TCSn:GFP* during LR development. Activity of cytokinin as visualized by *TCSn:GFP* is high in PCs on either side of LRP in wild-type plants. *TCSn:GFP* expression is reduced in PCs adjacent to LRP of the cytokinin-deficient *ipt3 5 7* mutant. White asterisks indicate borders of LRP or emerged LRs. Scale bar is 100 µM (this figure is available in colour at *JXB* online).

The LRP spacing defect phenotype was further analysed by counting the PC number between two closely positioned LRP in *ipt3 5 7* and *log4* roots compared to wild type. Fig. 3B shows that both cytokinin-synthesis mutants had a higher proportion of LRP separated by zero to three PCs (44.5% for *ipt3 5 7* and 47.9% for *log4*) as compared to wild type (22.8%). The largest difference in LRP specification between the cytokinin-synthesis mutants and the wild type was immediately adjacent to an already initiated LR and then declined gradually, showing an almost similar frequency at a distance of three PCs (Fig. 3B). This gradient of activity could possibly be due to a cytokinin flow originating in young LRP and caused by the combined activities of IPT5 and LOG4 that primarily affect the immediately adjacent cells (Fig. 2A). Fig. 4 shows that reduced cytokinin synthesis in and around LRP affects the cytokinin output signal in the zone of high cytokinin sensitivity surrounding LRP (Bielach *et al.*, 2012). The signal of the cytokinin reporter *TCSn:GFP* (Zürcher *et al.*, 2013) in wild type was high in cells neighbouring LRP but lower in these cells in roots of the cytokinin-synthesis mutant *ipt3 5 7*. This is consistent with a lower inhibitory activity of cytokinin on LR initiation in these cells.

To investigate whether the cytokinin receptor genes and response regulator genes are involved in the regulation of LR spacing, the double receptor mutants and type-B *ARR* mutants were analysed. A higher percentage of misplaced LRP as compared to wild type was formed in all three double receptor mutants, but the difference was not statistically significant (Fig. 3C). Among the type-B response regulator genes, the *arr1 12* (6.1±1.6%) and *arr10 12* (6.8±2.2%) double mutants showed significantly increased spacing defects compared to wild type (Fig. 3C). These data are consistent with the high degree of functional redundancy of cytokinin receptors and type-B *ARR* genes in regulating LR development (Chang *et al.*, 2013) and demonstrate that perception and transmission of the cytokinin signal is important to regulate LR spacing.

### *ACR4* expression is reduced in roots of cytokinin-deficient plants and the cytokinin status is lowered in *acr4* mutants

**Fig. 5.**
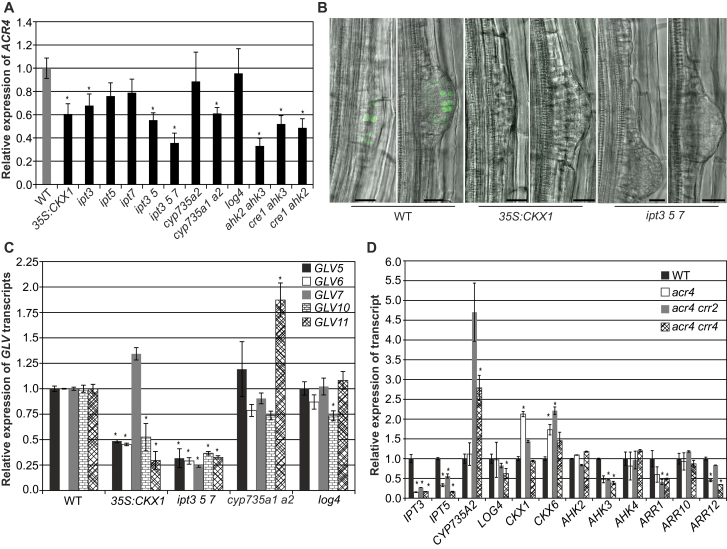
Interaction between the cytokinin and ACR4 signalling pathways on gene expression level. (A) *ACR4* expression analysis by qPCR in the roots of 11-d-old different cytokinin-deficient seedlings. (B) *ACR4::H2B:YFP* expression (green signals) is visible in LRP of wild type (WT) but is absent in cytokinin-deficient plants. Scale bar is 20 µM. (C) *GLV* gene expression analysis by qPCR in the roots of 11-d-old different cytokinin-deficient seedlings. (D) Transcript profiles of cytokinin metabolism and signalling genes in roots of 11-d-old *acr4* single and double mutants. Error bars represent SEM from three (A) or two (C, D) biological replicates. Each biological replicate contained roots from at least six individual plants. In all cases the expression level of wild type was set to 1 and the statistical significance of differences of expression values in mutants compared to wild type was determined by Student’s *t*-test (*P < 0.05) (this figure is available in colour at *JXB* online).

Because both the cytokinin and ACR4 pathways prevent neighbouring PCs from LRI, the relationship between these pathways was investigated. The expression of *ACR4* in roots of 11-d-old seedlings of cytokinin-synthesis and -signalling mutants was analysed. Fig. 5A shows that the abundance of the *ACR4* transcripts was lower in the roots of most plants with a reduced cytokinin status. The strongest reduction of *ACR4* transcript abundance (~60% reduction compared to wild type) was noted in *ipt3 5 7* triple mutants. Further, in wild type, *pACR4*-driven *H2B:YFP* expression was observed in the LRP while fluorescence of YFP was hardly detectable in the cytokinin-deficient *35S:CKX1* and *ipt3 5 7* background (Fig. 5B). However, *ACR4* transcript analysis at different time points following exogenous addition of cytokinin did not reveal a strong influence of cytokinin on the steady-state level of *ACR4* mRNA (Brenner *et al.*, 2012 and data not shown). Taken together, this shows that *ACR4* expression is reduced in cytokinin-deficient plants but that *ACR4* is not a primary cytokinin response gene. The reduced LR spacing in cytokinin-deficient plants might be partly accomplished through lowered expression of *ACR4*.

The transcript levels of the *GLV* genes, which encode small signalling peptides that are the putative ligands of ACR4 (Meng *et al.*, 2012; Fernandez *et al.*, 2013), were then investigated. The expression of all five *GLV* genes that are specifically expressed during early stages of LR development (Fernandez *et al.*, 2013) showed a lower expression in the roots of plants with a globally reduced cytokinin status (Fig. 5C), even though these form more LRP. The strongest reduction of *GLV* gene transcript levels (~70–80% as compared to wild type) was again observed in *ipt3 5 7* triple mutant plants, similar to the strong reduction of *ACR4* (Fig. 5A,C). However, in contrast, the *cyp735a* double mutant and the *log4* mutant did not display strong changes of *GLV* gene expression compared to wild type. It could be that changes in *t*Z do not affect *GLV* gene expression and that the local changes of the cytokinin content expected in *log4* mutants are diluted in whole root extracts. In any case, cytokinin appears to positively regulate *GLV* gene expression in roots. However, whether this results in altered ACR4 activity that affects LRI remains to be shown.

The transcript profiles of cytokinin genes were analysed by qPCR in the roots of *acr4* mutants, and in double mutants combining *acr4* with mutant alleles of two other family members, *CRR2* and *CRR4* (De Smet *et al.*, 2008). The analysis showed significantly changed transcript levels for several of the cytokinin genes. *IPT3* and *IPT5* were both downregulated in the *acr4* mutants while *CKX1* and *CKX6* were upregulated (Fig. 5D). Similarly, several genes encoding cytokinin signalling factors were downregulated, including the receptor gene *AHK3* and the type-B *ARR* genes *ARR1* and *ARR12*. Together, the altered expression of cytokinin genes suggests a lower cytokinin status in *acr4* mutant roots. Interestingly, *CYP735A2* was strongly upregulated in the *acr4 ccr2* and *acr4 crr4* double mutants, indicating a negative control of the ACR4 system on the synthesis of *t*Z-type cytokinins (Fig. 5D).

### Cytokinin and ACR4 inhibit the initiation of neighbouring PCs through independent non-hierarchical pathways

**Fig. 6.**
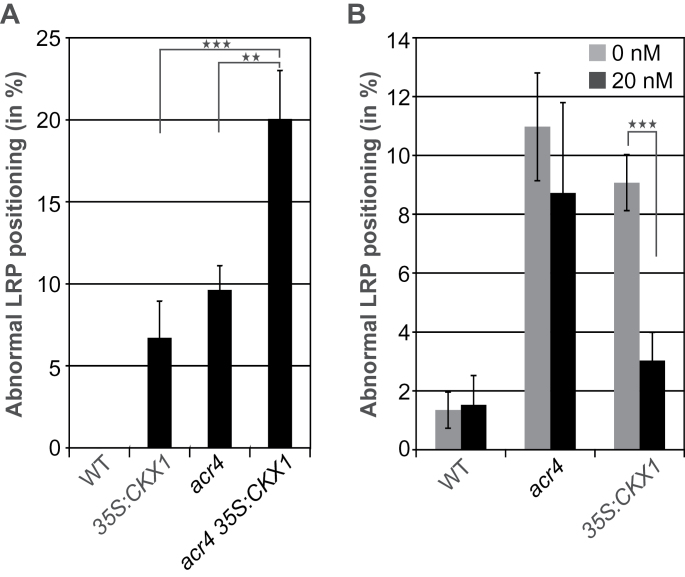
Effects of a lowered cytokinin status and *ACR4* mutation on LR spacing are additive. (A) Percentage of immediately adjacent LRP in 11-d-old roots of wild type (WT), *35S:CKX1*, *acr4*, and *acr4 35S:CKX1* hybrids. n = 15–18. (B) Misplacement of LRP was partially supplemented by exogenous cytokinin (BA) in *35S:CKX1* plants but not in *acr4*. Seedlings were grown for 11 d on medium with or without 20nM BA prior to analysis. n = 15 for wild type, 32 for *acr4*, and 15 for *35S:CKX1*. Error bars in (A, B) indicate SEM. The statistical significance of differences was calculated using two-tailed Student’s *t*-test (**P < 0.01; ***P < 0.001).

To further investigate whether cytokinin and ACR4 control the positioning of LR through the same or separate pathways, the LR spacing phenotype of the *acr4 35S:CKX1* hybrid seedlings was compared to the parental phenotypes. The hybrid *acr4 35S:CKX1* plants displayed a much higher percentage of aberrantly positioned LRP (20.0±2.9%) than both parents (Fig. 6A). This largely additive effect suggests that cytokinin and ACR4 function at least partly in separate pathways in preventing neighbouring PCs from LRI. Consistently, addition of cytokinin to the growth medium had only a weak effect on the LR spacing defect in *acr4* mutants, while it did lower the LR spacing defect by 67% in *35S:CKX1* seedlings (Fig. 6B).

## Discussion

**Fig. 7.**
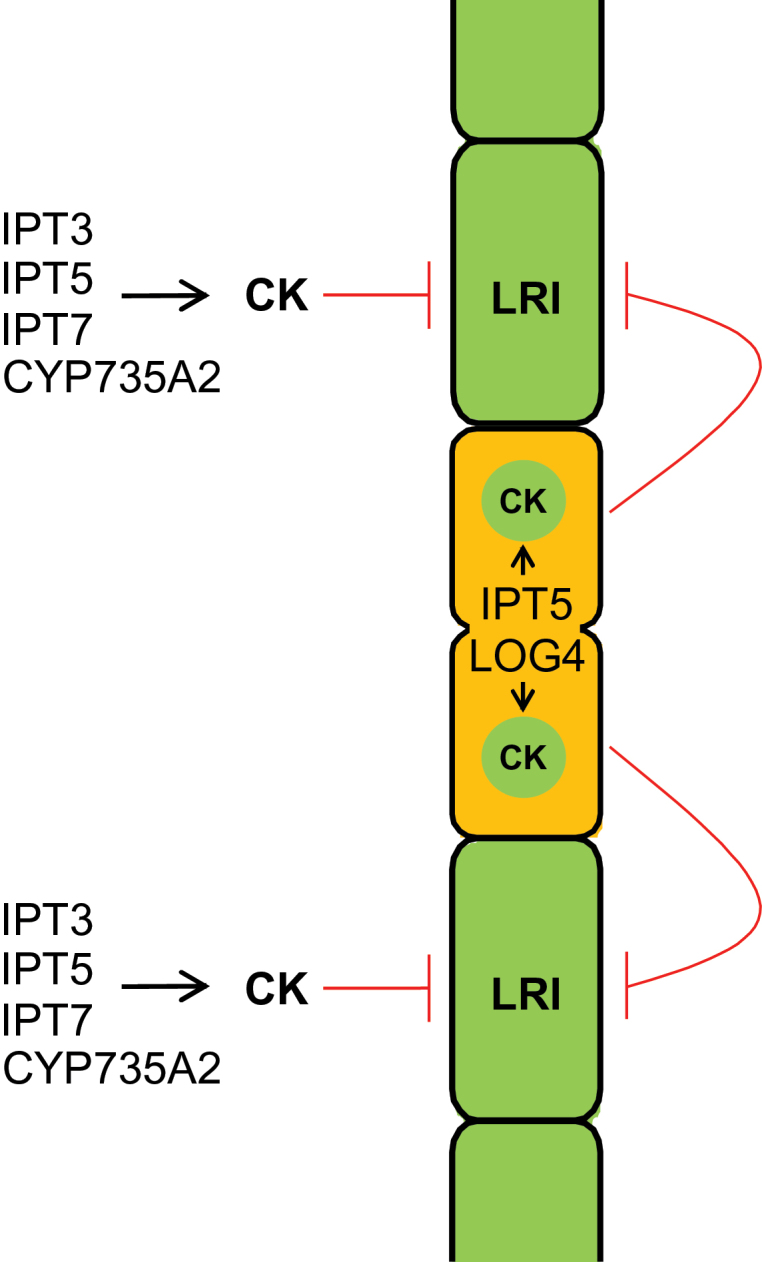
Model illustrating the action of cytokinin in regulating LR spacing. The model predicts that the suppression of LRI in PCs neighbouring existing LRFCs is a combined effect of cytokinin synthesized in LRFCs and cytokinin synthesized by neighbouring cells. Activity of IPT5 and LOG4 in LRFCs causes the formation of iP, which diffuses laterally. Neighbouring cells convert iP to *t*Z by CYP735A2 and, in addition, synthesize their own cytokinin to inhibit LR formation. LRFCs are characterized by a high auxin-to-cytokinin ratio, indicated by the orange colour, while adjacent PCs are characterized by a low auxin-to-cytokinin ratio, indicated in green (this figure is available in colour at *JXB* online).

The positioning of LRP along the *Arabidopsis* main root defines a usually regular rhizotactic pattern and LRP do not form in close proximity to each other. The hormone cytokinin functions in suppressing the formation of LRP close to existing ones and thus acts as a positional cue regulating the distance between LRP. The positional signal is generated by locally and developmentally controlled precise tuning of cytokinin synthesis gene expression. The lower distance of LRP in cytokinin-synthesis mutants is hypothesized to be due to a local inhibitory cytokinin gradient originating from the LRFCs, and to cytokinin produced in PCs neighbouring LRFCs. This paracrine action of cytokinin, a combination of non-cell autonomous and cell autonomous activity, inhibits the formation of LRP in PCs neighbouring existing LRP. This hypothesis is supported by (i) the activation of cytokinin synthesis genes early during LRP development and their expression in neighbouring PCs; (ii) the fact that disruption of these genes causes aberrant positioning of LRP, with the strongest effect in the immediate vicinity of existing LRP; and (iii) a gradual decrease of the inhibitory cytokinin action in wild type with increasing distance from LRFCs. The proposed activity of cytokinin is summarized in a model (Fig. 7).

The expression pattern of cytokinin synthesis genes showed that *IPT5* and *LOG4* were expressed very early during LR initiation (Fig. 2) (Miyawaki *et al.*, 2004; Kuroha *et al.*, 2009). However, *TCS:GFP* reporter expression revealed that cytokinin signalling is strongly repressed during early phases of LR development (Bielach *et al.*, 2012), indicating that the signal generated by IPT5 and LOG4 in the initiated LR was suppressed or diffused laterally. Considering the strong enhancement of abnormal LR positioning when *IPT5* is mutated in addition to *IPT3* and *IPT7* and, in particular, the LR positioning defect caused by *LOG4* mutation alone (Fig. 3A,B), it is possible that cytokinin synthesized by IPT5 and LOG4 in the LRFCs is transmitted to neighbouring PCs to prevent LRI. This is in agreement with the lowest frequency of new LRP immediately adjacent to existing ones in wild type and their gradually increasing frequency with increasing distance from existing LRP (Fig. 3B). Consistently, the strongest increase of aberrantly positioned LRP in *ipt3 5 7* and *log4* mutants was found immediately adjacent to existing ones (Fig. 3B). Further, expression of a cytokinin-degrading *CKX* gene in the LRFCs reduces the distance from new neighbouring LRP, which is in line with a suppressive function of LRFC-derived cytokinin in neighbouring cells (Laplaze *et al.*, 2007). In accordance with the idea of a cytokinin signal coming from LRFCs was the finding that a cytokinin output signal is detected few hours after LRI in adjacent cells (Bielach *et al.*, 2012). However, the authors did not report any cell-to-cell movement of a cytokinin signal. An alternative possibility to explain the gradual difference in response of PCs neighbouring LRFCs to a suppressive cytokinin signal would be differences in their cytokinin sensitivity. However, a strongly enhanced percentage of LRP immediately adjacent to existing LRP was not found in mutants retaining single cytokinin receptors (Fig. 3C), although they generally show a higher LRP and LR density (Chang *et al.*, 2013). In any case, there is an additional relevant role of cytokinin synthesis genes (*IPT3*, *IPT7*) expressed in pericycle tissue adjacent to existing LRP. This argues for a combined action of LRP-derived cytokinin and cytokinin synthesized in neighbouring cells (Fig. 7).

Recently, other cases of cytokinin action over a short distance involving LOG4 have been reported. Cytokinin production by LOG4 in embryonic xylem precursor cells has been shown to induce procambial cell proliferation in neighbouring cells, thus regulating growth and patterning during vascular development in a non-cell-autonomous manner (De Rybel *et al.*, 2014; Ohashi-Ito *et al.*, 2014). In addition, a computational model has demonstrated that cytokinin generated by LOG4 may signal over several cell layers in the shoot apical meristem and act together with CLV signalling as a positional cue within the stem cell niche (Chikarmane *et al.*, 2012). This indicates that the final activating step during cytokinin biosynthesis has a pivotal role in establishing local cytokinin gradients and, more specifically, suggests that LOG4 has an important role in cellular patterning.

The analysis of *cyp735a* mutants indicated that *t*Z has strong activity in suppressing the formation of LRP. This reveals a novel local function of the *CYP735A* genes known for their importance in synthesizing *t*Z-type cytokinin for root-to-shoot communication (Kiba *et al.*, 2013). *CYP735A2* promoter-driven reporter gene activity was present in the cells surrounding the LRP but not in the LRP itself. *CYP735A2* is one of the genes most strongly downregulated by auxin in an ARF7/ARF19-dependent manner in the root (Parizot *et al.*, 2010). Unfortunately, due to the low expression of *CYP735A1*, there was no similar information to evaluate its behaviour in this context. However, it could be that iP synthesized by IPT5 and LOG4 in LRFC reaches neighbouring cells by diffusion and/or transport and is then converted to *t*Z.

How could the activity of cytokinin and the regulation of cytokinin synthesis gene be linked to auxin, which is the master regulator of LR development? Some indications come from gene expression studies. The expression of *IPT5* is activated during LR formation in an ARF7/ARF19-dependent manner (Parizot *et al.*, 2010) and the *LOG4* promoter has been shown to be a direct target of the TMO5-LHW transcription factors acting downstream of the BDL/IAA12-MP/ARF5 auxin response module (De Rybel *et al.*, 2014), acting a bit later than ARF7-ARF19 in LRI (De Smet *et al.*, 2010). Interestingly, the *mp* mutant shows a LR spacing defect (De Smet *et al.*, 2010) that might be partially due to reduced *LOG4* expression. Additional cytokinin genes that are also switched on through the ARF7-ARF19 and TMO5-LHW pathways are two auxin-responsive *CKX* genes (*CKX1*, *CKX6*) (Parizot *et al.*, 2010) and the auxin-responsive negative regulator *AHP6* (Ohashi-Ito *et al.*, 2014), which is expressed in LRs from the early stages onwards (Moreira *et al.*, 2013). Activation of these genes in the LRFCs could maintain a low cytokinin status in these cells to avoid a negative impact of cytokinin on their further development. Indeed, occasional periclinal cell division in the LRFC of *ahp6* mutants suggests a repressive action of AHP6 on cytokinin signalling in these cells (Moreira *et al.*, 2013). Taken together, the auxin-induced lowering of the cytokinin status in the LRFCs and cytokinin synthesis in neighbouring cells would contribute to a high auxin/low cytokinin ratio in LRFCs, and a low auxin/high cytokinin ratio in the neighbouring cells. Comparisons of *DR5* and *TCS* reporter signals have consistently indicated that auxin and cytokinin response maxima are complementary in the pericycle and during LR development (Benková *et al.*, 2003; Bielach *et al.*, 2012).

A strongly lowered cytokinin status does not release the inhibition of all xylem pole PCs that are developmentally primed to eventually become LRFCs, suggesting that one or several other systems operate in parallel to control LR spacing. One such system is based on ACR4. Expression of *ACR4* was lower in the roots of cytokinin-deficient plants, and *acr4* mutants had a lower cytokinin status (Fig. 5). A genetic analysis did not reveal a hierarchical order between the cytokinin and the ACR4 pathway (Fig. 6). However, the reciprocal influence on transcript levels indicates that crosstalk exists and suggests that ACR4 has a positive impact on the cytokinin pathway and *vice versa*. This reciprocal influence might be gradual and not necessarily strongly influence the outcome of the genetic analysis, although the percentage of misplaced LRP was higher than it would be from an additive effect in the *acr4 35S:CKX1* hybrids (Fig. 6A). Moreover, the extent of the local reciprocal influence on the transcript levels could not be evaluated as these were measured in whole root extracts. Regardless, one pathway could act through transcriptional regulation in a feed forward-like manner to enhance the other pathway’s suppression of LRI in neighbouring cells.

Together, the results show the involvement of cytokinin in regulating root system architecture soon after founder cell selection, the first key step in LR formation from a primed cell. Cytokinin is proposed to act locally as a paracrine signal that originates in LRFCs and its neighbouring cells, acting both as a suppressor of LRI and to determine which of the competent sites develop into LR. An instructive signal from LRFCs to neighbouring PCs for LRI would spare resources and help develop an optimal root system. Furthermore, cytokinin may act as an endogenous signal responding to environmental cues such as nutrients and osmotic conditions (Malamy *et al.*, 2005; Ramireddy *et al.*, 2014). In this way it contributes to the developmental plasticity of root architecture, which has to adapt constantly to a highly complex subterranean environment.

## Supplementary data

Supplementary data are available at *JXB* online.

Fig. S1. Spatio-temporal expression of selected cytokinin metabolism genes during LR development.

Fig. S2. LR spacing is altered in mutants with a lower cytokinin status.

Table S1. Primers used for quantitative real-time reverse transcription PCR.

Supplementary Data

## Abbreviations:

LR: lateral root
LRP: lateral root primordium
LRFC: lateral root founder cell
PC: pericycle cell
BA: 6-benzylaminopurine
tZ: *trans*-zeatin.
