# Supplementary material for: Cytokinin as a positional cue regulating lateral root spacing in Arabidopsis
Source: J Exp Bot. 2015 May 27;66(15):4759–68. doi: 10.1093/jxb/erv252 (PMC4507779; doi:10.1093/jxb/erv252)
Supplement: Supplementary Data [file supp_66_15_4759__index.html]

Cytokinin as a positional cue regulating lateral root spacing in Arabidopsis — Cytokinin as a positional cue regulating lateral root spacing in Arabidopsis — Supplementary Data 

# Cytokinin as a positional cue regulating lateral root spacing in *Arabidopsis*

## Supplementary Data

Data files

- Supplementary Data - Supplementary Data
