# Supplementary material for: Cytokinin as a positional cue regulating lateral root spacing in Arabidopsis
Source: J Exp Bot. 2015 May 27;66(15):4759–68. doi: 10.1093/jxb/erv252 (PMC4507779; doi:10.1093/jxb/erv252)
Supplement: Supplementary Data [file supp_erv252_jexbot148445_file001.pdf]

Cytokinin as a positional cue regulating lateral root spacing in Arabidopsis

Ling Chang, Eswarayya Ramireddy and Thomas Schmülling

Supplementary Data

Figure S1. Spatio-temporal expression of selected cytokinin metabolism genes during lateral root development.

Figure S2. Lateral root spacing is altered in mutants with a lower cytokinin status.

Table S1. Primers used for quantitative real-time RT-PCR.

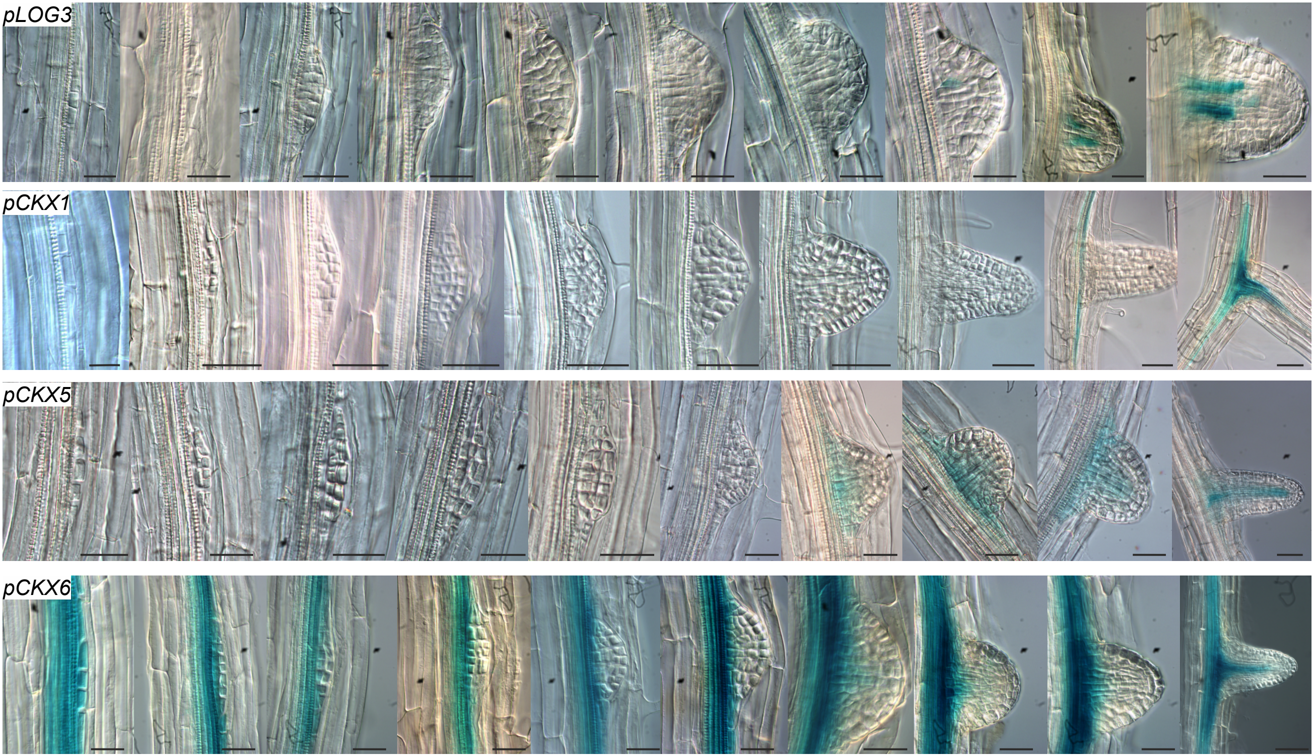

Figure S1. Spatio-temporal expression of selected cytokinin metabolism genes during lateral root development. Staining pattern of reporter genes are shown from left to right starting with stage I LRP to emerged LR. The respective promoter is indicated in the upper left corner of each picture series. Pictures showing GUS analysis were obtained from 3-d-old seedlings. *CKX6:GUS* seedlings were stained with GUS reaction buffer for 1 hour and cleared. All other seedlings harboring promoter:GUS reporter genes were incubated overnight before clearance. Scale bars are 50 µM.

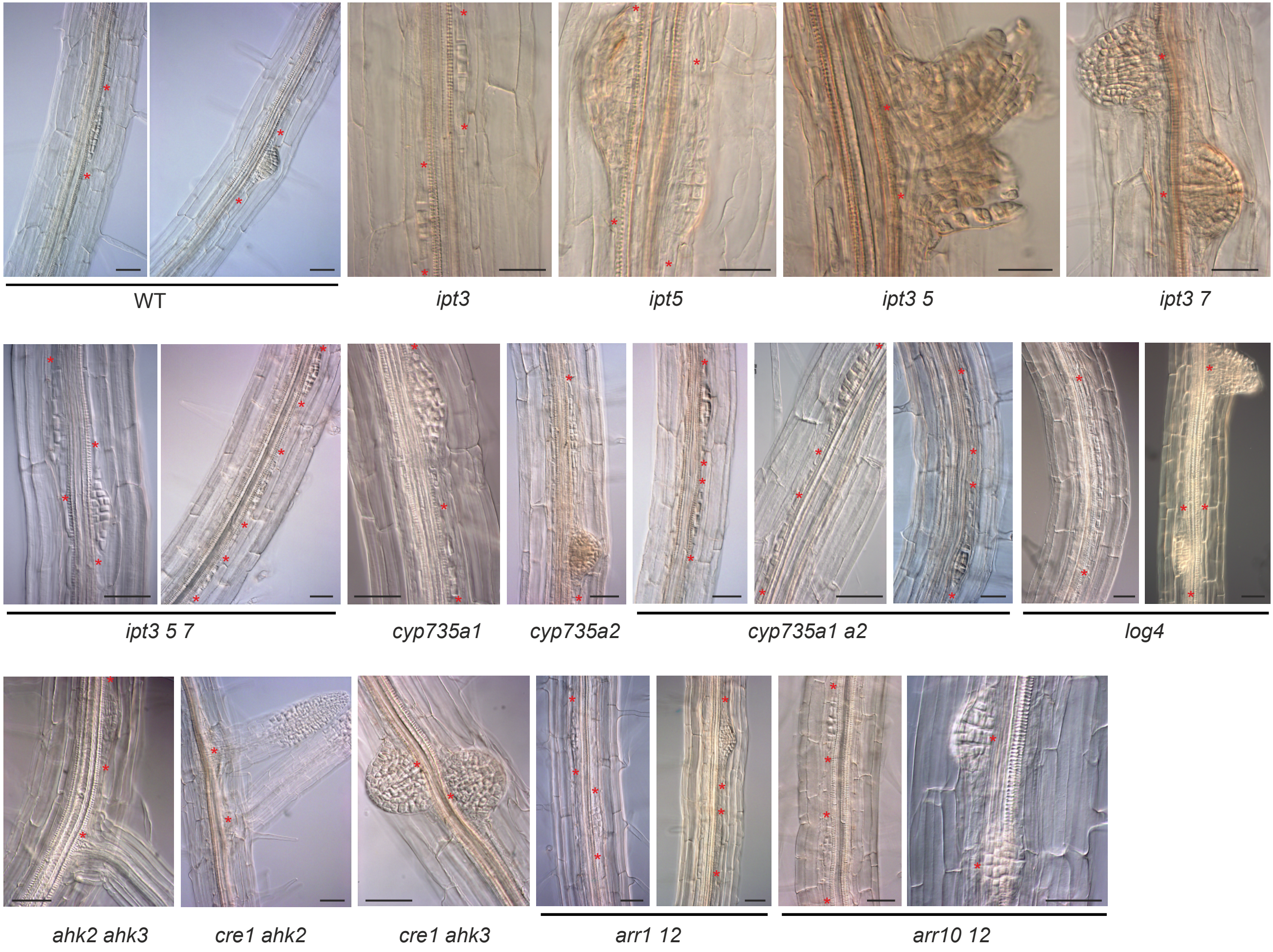

Figure S2. Lateral root spacing is altered in mutants with a lower cytokinin status. Examples of misplaced LRP and LR in different mutants of cytokinin synthesis and signaling genes are shown. No proximal LRP and LR were observed in wild type. Red asterisks indicate borders of LRP or emerged LR. Scale bars are 50 μM.

Supplemental Table 1. Primers used for quantitative real-time RT-PCR.

Gene name	Primer sequence
UBC10	5‘-CCATGGGCTAAATGGAAA-3‘
	5‘-TTCATTTGGTCCTGTCTTCAG-3‘
PDF1	5‘-CCATTAGATCTTGTCTCTCTGCT-3‘
	5‘-GACAAAACCCGTACCGAG-3‘
ACR4	5‘- TTCAACTGCGCGCTGTCCTC-3‘
	5‘- CACTCGCGATCCAACCCGT-3‘
IPT3	5‘-TCCGTCCTAAACCGTGGAA-3‘
	5‘-CCATTCCACTCTCCACCATC-3‘
IPT5	5‘-CTTGGGATAACTCAGTGGCT-3‘
	5‘-CTCCGGTAGGAGAATATTGG-3‘
CKX1	5‘-ACGACCCTCTAGCGATTCT-3‘
	5‘-CGGCAGTATTGATGCGTA-3‘
CKX6	5‘-CAATACGCACCAACCAAG-3‘
	5‘-CCTATTGGGCCTGAAAGA-3‘
LOG4	5‘-TGGAGGTCAACAATGAAACCA -3‘
	5‘-GCGAAACCAAACCCATCAAA -3‘
CYP735A2	5‘-GGTTGTTCCGTATCTCTCACC -3‘
	5‘-CGTCTTGGTGTGAGGAAGTAG -3‘
AHK2	5‘-GAGCTTTTTGACATCGGG-3‘
	5‘-TTCTCACTCAACCAGACGAG-3‘
AHK3	5‘-GTGACCAGGCCAAGAACTTA-3‘
	5‘-CTTCCCTGTCCAAAGCAA-3‘
AHK4	5‘-CCATCTTGCGGAACAATC-3‘
	5‘-GAATCCCAGCTATTCCGAC-3‘
ARR1	5‘-CATCACGGGCAACAACCCCA-3‘
	5‘-GCAAAGACTGCTGACCCGCT-3‘
ARR10	5‘-CCTGACACCTTGGAATGGA-3‘
	5‘-GTCACTGGACCTGAGTTGTT-3‘
ARR12	5‘-ACCGCCCAATCCCTGTTGGA-3‘
	5‘-GAAAGGCTGCGGACACCGAT-3‘
GLV5	5‘-GGTGATAAGGATGAAGTGCTAAGTG-3‘
	5‘-AGTTGTTTTTGGGAGGATGGTG-3‘
GLV6	5‘-CCCTACTCATCTCCTAGCCA-3‘
	5‘-GACTCGAAGTTGTTCGCTTG-3‘
GLV7	5‘-GTTTCTCCTTTCGCTATTCACTTC-3‘
	5‘-ATCATCAGTTTTCGTCCTCGT-3‘
GLV10	5‘-TACAGCGTCTCGGTTTTCAC-3‘
	5‘-TCAGTTATGGCGTGGAGG-3‘
GLV11	5‘-TGGTACCCAAATTGCCAAGA-3‘
	5‘-AGGGTTGCTATAATCGGCAG-3‘
